# Prediction and Dissection of Widely-Varying Association Rate Constants of Actin-Binding Proteins

**DOI:** 10.1371/journal.pcbi.1002696

**Published:** 2012-10-04

**Authors:** Xiaodong Pang, Kenneth H. Zhou, Sanbo Qin, Huan-Xiang Zhou

**Affiliations:** 1 Department of Physics and Institute of Molecular Biophysics, Florida State University, Tallahassee, Florida, United States of America; 2 Lawton Chiles High School, Tallahassee, Florida, United States of America; University of Virginia, United States of America

## Abstract

Actin is an abundant protein that constitutes a main component of the eukaryotic cytoskeleton. Its polymerization and depolymerization are regulated by a variety of actin-binding proteins. Their functions range from nucleation of actin polymerization to sequestering G-actin in 1∶1 complexes. The kinetics of forming these complexes, with rate constants varying at least three orders of magnitude, is critical to the distinct regulatory functions. Previously we have developed a transient-complex theory for computing protein association mechanisms and association rate constants. The transient complex refers to an intermediate in which the two associating proteins have near-native separation and relative orientation but have yet to form short-range specific interactions of the native complex. The association rate constant is predicted as *k*_a_ = *k*_a0_ 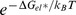, where *k*_a0_ is the basal rate constant for reaching the transient complex by free diffusion, and the Boltzmann factor captures the bias of long-range electrostatic interactions. Here we applied the transient-complex theory to study the association kinetics of seven actin-binding proteins with G-actin. These proteins exhibit three classes of association mechanisms, due to their different molecular shapes and flexibility. The 1000-fold *k*_a_ variations among them can mostly be attributed to disparate electrostatic contributions. The basal rate constants also showed variations, resulting from the different shapes and sizes of the interfaces formed by the seven actin-binding proteins with G-actin. This study demonstrates the various ways that actin-binding proteins use physical properties to tune their association mechanisms and rate constants to suit distinct regulatory functions.

## Author Summary

Actin polymerization and depolymerization drive cell motility and are regulated by a variety of actin-binding proteins. The widely-varying rate constants (*k*_a_) of the actin-binding proteins associating with G-actin, spanning at least three orders of magnitude, appear to be tuned for their distinct regulatory functions. Here we applied our previously developed transient-complex theory to study the association kinetics of seven actin-binding proteins with G-actin. These proteins exhibit three classes of association mechanisms, due to their different molecular shapes and flexibility. The 1000-fold *k*_a_ variations among them can mostly be attributed to disparate inter-protein electrostatic interactions. By computing the association mechanisms and quantifying the physical determinants of association rate constants, the present study reveals critical links between the structure and function of the actin-binding proteins.

## Introduction

Actin is an abundant protein that constitutes a main component of the eukaryotic cytoskeleton. Actin polymerization and depolymerization drive essential cellular processes such as cell motility. Nucleation, growth, and disassembly of actin filaments allow cells to rapidly respond to external stimuli. It is known that addition of actin monomers at the barbed end of actin filaments is diffusion-limited [1] and assisted by electrostatic interactions [2]. Actin dynamics is regulated by a variety of actin-binding proteins (ABPs). The functions of ABPs include the nucleation of actin polymerization, promotion of nucleotide exchange in G-actin, sequestration of G-actin, and severance and capping of actin filaments. Many of these functions involve formation of 1∶1 complexes with G-actin. The kinetics of forming these complexes undoubtedly is critical to the regulatory functions of the ABPs. Many of the bimolecular rate constants have been determined experimentally [3]–[9], and the values cover at least three orders of magnitude. Recently we have developed a method for computing protein association mechanisms and rate constants, and applications to a set of 49 complexes, including two ABP:G-actin complexes, showed that the calculated rate constants are highly accurate [10]. Here we carried out a systematic computational study on the actin-association kinetics of seven ABPs in order to gain better understanding on how their regulatory functions are linked to their structures.

**Figure 1:**
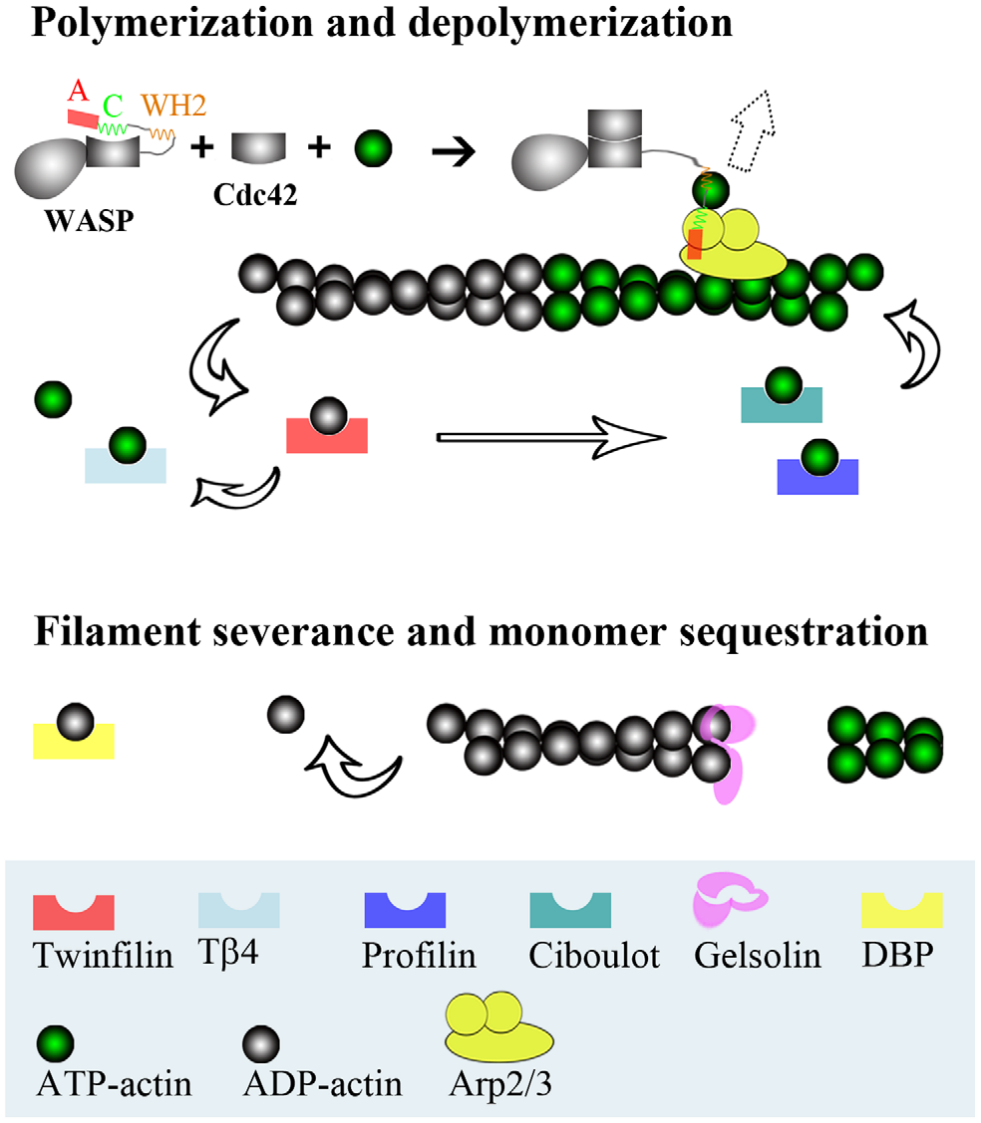
Regulatory functions of ABPs. Auto-inhibited WASP is activated by binding Cdc42, and can then work with the Arp2/3 complex to nucleate new actin filaments (direction indicated by a dashed block arrow). By forming 1∶1 complexes with G-actin, ABPs like profilin, ciboulot, Tβ4, twinfilin, and DBP prevent spontaneous nucleation. With these 1∶1 complexes, some (profilin and ciboulot) still allow addition at the barbed end of actin filaments, and others (Tβ4, twinfilin, and DBP) just sequester G-actin. Gelsolin servers and then caps the barbed end of actin filaments.

The seven ABPs studied here span a range of regulatory functions (Figure 1). The Wiskott-Aldrich syndrome protein (WASP) stimulates the actin nucleation activity of the Arp2/3 complex. This function of WASP resides in the C-terminal region (hereafter referred to as WCA). It is believed that addition of a G-actin molecule, recruited by the WASP WCA, to the Arp 2 and Arp3 subunits of the Arp2/3 complex, creates the nucleus for a new actin filament [11], [12]. Filament growth occurs only when G-actin is present above a “critical” concentration. Typically, the barbed end grows with the addition of ATP-G-actin; ATP is then hydrolyzed on the filament, and the pointed end shrinks with the departure of ADP-G-actin.

In the cytoplasm, G-actin is sequestered by ABPs such as profilin, ciboulot, thymosin β4 (Tβ4), and twinfilin; the first three favor ATP-G-actin [8], [13], [14] whereas the last favors ADP-G-actin [7]. The total G-actin pool is much higher than the critical concentration. G-actin sequestered by Tβ4 and twinfilin is incapable of adding to the barbed end of actin filaments, but ATP-G-actin bound to profilin and ciboulot is as competent as free ATP-G-actin for filament growth at the barbed end. The rapid exchange of G-actin molecules among these monomer-sequestering proteins, along with the promotion of the exchange of ATP for ADP in G-actin by profilin, ensures a proper portion of G-actin ready for filament growth [15]. Actin filaments can be severed and capped at the new barbed end by gelsolin. Actin can be released by tissue injury or cell death to the bloodstream, where polymerization is lethal. This ill fate is prevented by the severing and capping function of a plasma isoform of gelsolin, in conjunction with monomer sequestration by vitamin-D binding protein (DBP).

**Figure 2:**
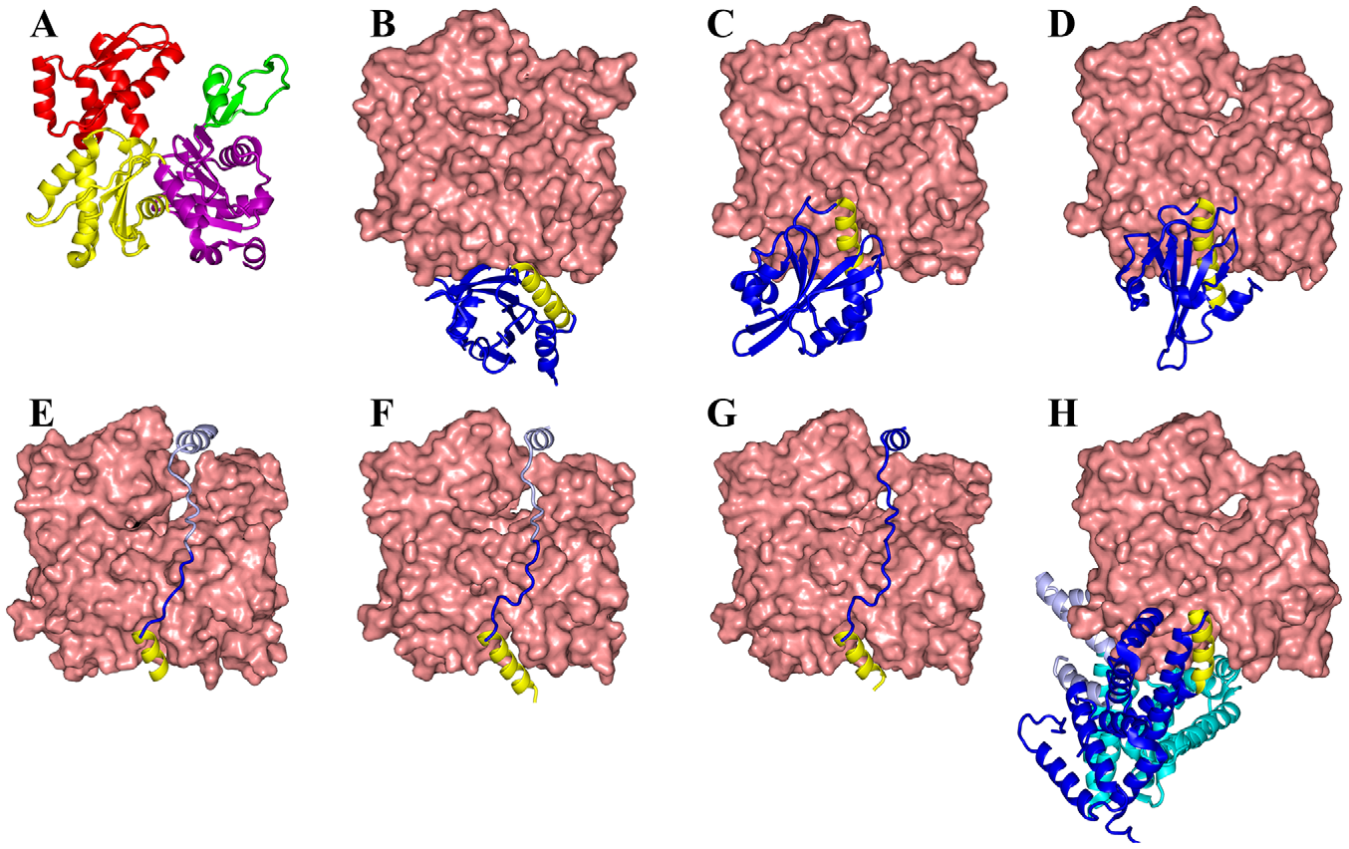
Structures of seven ABPs complexed with G-actin. (A) G-actin, with subdomains shown in magenta, green, yellow, and red, respectively. (B) Profilin. (C) Twinfilin ADF-homology domain 2. (D) Gelsolin domain 1. (E) WASP WH2, linker, and central segments. (F) Ciboulot domain 1. (G) Thymosin β4. (H) Vitamin-D binding protein. The cleft-lying helices of all seven ABPs are shown in yellow. The C-terminal portions of the ABPs in (E) and (F), shown in light color, are modeled and can easily dissociate. In (H) the three domains of DBP are in dark blue, cyan, and light blue respectively.

The seven ABPs all bind to the barbed end of G-actin, each with a helix lying in the cleft between subdomains 1 and 3 [8], [16]–[21] (Figure 2). The cleft-lying helices in six of these structures lie in approximately the same position, while that of profilin is more to the back of the G-actin molecule. In WASP WCA, ciboulot domain 1, and Tβ4, the cleft-lying helices run from the back to the front of the G-actin molecule, but the direction of the helices is reversed in the other four ABPs. Beyond the cleft-lying helices, the seven ABPs span a significant range of structural diversity. The twinfilin C-terminal actin-depolymerizing factor homology domain (ADF-H 2) and gelsolin domain 1 dock to G-actin from the front, whereas profilin docks from the base. WASP WCA, ciboulot domain 1, and Tβ4 are disordered in the free state [6], [8], [22]–[24] and form extended structures upon binding G-actin, hanging over the latter's nucleotide-binding cleft (which separates the subdomains 1 and 2 from subdomains 3 and 4). Finally the three domains of DBP engulf G-actin tightly from the front, base, and back, respectively. Given their structural diversity, it can be anticipated that the ABPs exhibit a variety of association mechanisms and a range of association rates.

Recently we developed the transient-complex theory for calculating protein association mechanisms and association rate constants [10], [25]. Two proteins reach a transient complex by translational and rotational diffusion, and then form the final, native complex by conformational rearrangement. In the transient complex, the two proteins have near-native separation and relative orientation, but have yet to form most of the stereospecific native interactions. When the conformational rearrangement is fast, the whole association process is rate-limited by the diffusional approach to the transient complex. The rate constant can then be calculated as
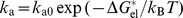
where *k*_a0_ is the basal rate constant, i.e., the rate constant for reaching the transient complex by free diffusion, and the Boltzmann factor captures the bias of inter-protein electrostatic interactions. We have demonstrated that, by adaptively applying the transient-complex based approach, we can study the association of not only relatively rigid proteins but also intrinsically disordered proteins and proteins whose breathing motions are essential for accommodating the incoming partners [10].

Our large-scale application of the transient-complex based approach to demonstrate its prediction accuracy happened to include two ABPs: profilin and gelsolin domain 1 [10]. They piqued our interest in the structure-function relations of ABPs in general, in particular the roles of their actin-association kinetics in linking structure and function. The present study was aimed at elucidating these roles by computing the association mechanisms and quantifying the physical determinants of association rate constants. The results demonstrate the versatility of ABPs in using molecular flexibility and surface charges to tune association mechanisms and rate constants to suit distinct regulatory functions.

## Results

**Figure 3:**
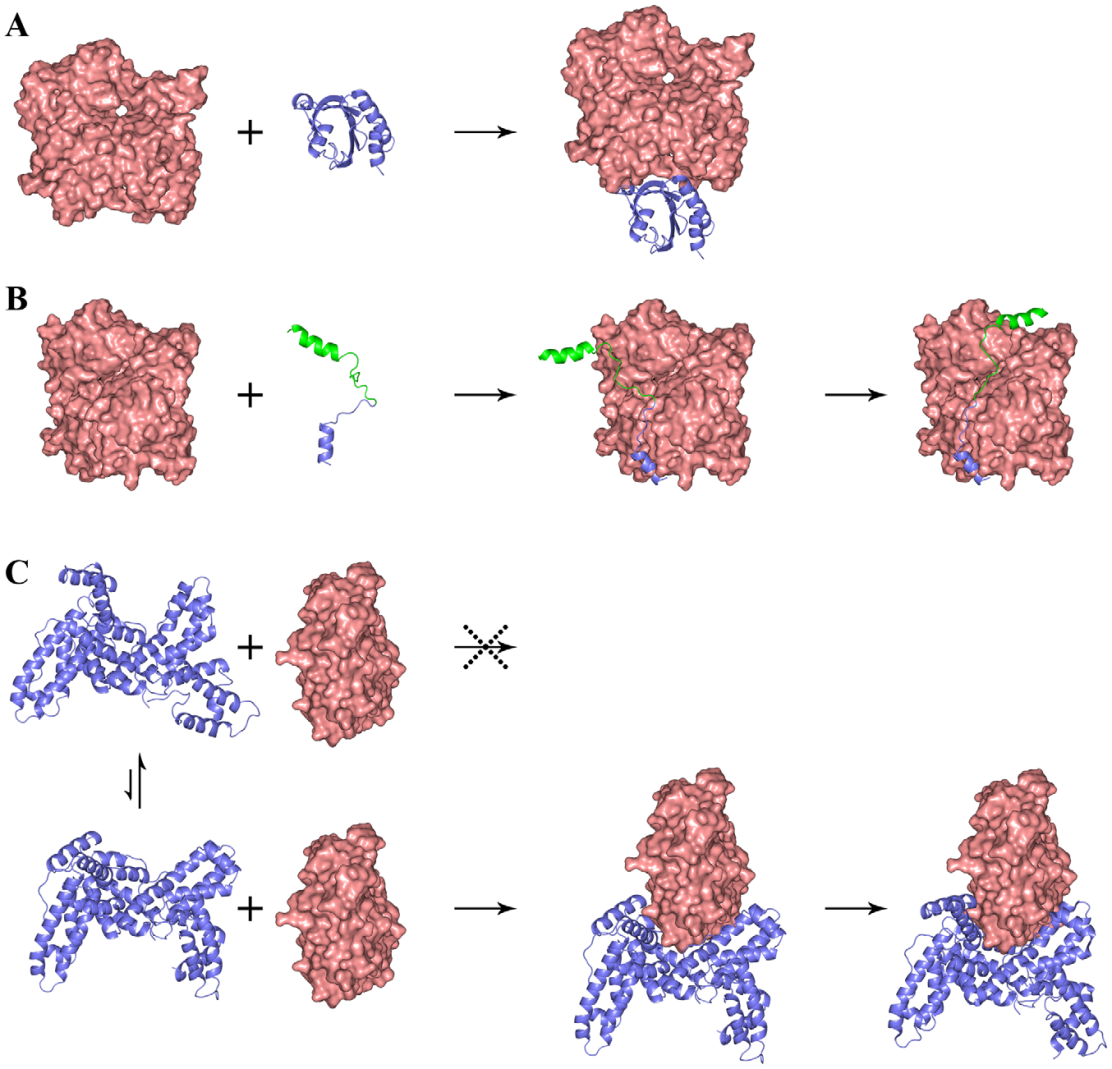
Three classes of association mechanisms. (A) A relatively rigid globular ABP like profilin reaches the transient complex (not shown) with G-actin by diffusion and then forms the contacts nearly all at once to produce the native complex. The binding of twinfilin ADF-homology domain 2 and gelsolin domain 1 to G-actin follows the same mechanism. (B) The dock-and-coalesce mechanism for the G-actin binding of WASP WCA, an intrinsically disordered protein. (C) Vitamin-D binding protein has an opening between its domains 1 and 3, in both the free state and the G-actin-bound state, that is too narrow for G-actin to enter. So DBP must undergo breathing motion to transiently widen its opening. Once G-actin is inside, the opening quickly narrows to clamp around G-actin. In (A) G-actin has the orientation as in Figure 1; in (B) it is rotated clockwise (viewed from top) around a vertical axis by 45°; and in (C) it is rotated counterclockwise (viewed from top) around a vertical axis by 90°.

Our transient-complex based calculations show that the seven ABPs exhibit three different classes of association mechanisms (Figure 3). Profilin, twinfilin ADF-H 2, and gelsolin domain 1 are relatively rigid globular domains. Their association with actin is accompanied by minimal backbone motions. WASP WCA, ciboulot domain 1, and Tβ4 are intrinsically disordered [6], [8], [22]–[24] and undergo disorder-to-order transitions upon binding G-actin to form extended structures. Their association follows a dock-and-coalesce mechanism, first proposed for the binding of another intrinsically disordered protein [10]. The three domains of DBP are organized into a fork, which engulfs G-actin from three directions. In both the free and bound conformations [18] the opening between domain 1 and domain 3 is too narrow for G-actin to enter. Therefore during the binding process DBP must undergo a breathing motion to transiently widen the opening for G-actin to enter. Once G-actin is inside, domains 1 and 3 of DBP tighten their clamp on G-actin.

The computed association rate constants of the seven ABPs are listed in Table 1, along with the experimental data and the ionic strengths of the measurements. Overall there is good agreement between computation and experiment. In our transient-complex based computation, the association rate constant is teased out into a basal rate constant, *k*_a0_, and an electrostatic contribution, 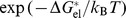. This teasing out provides us with a better handle on quantifying the physical determinants of the rate constant. The basal rate constants calculated for the seven ABPs range from 7.4×10^4^ M^−1^ s^−1^ for DBP to 2.5×10^6^ M^−1^ s^−1^ for WASP WCA (Table 1), conforming to the relatively narrow spread of *k*_a0_ found previously for 132 complexes [10]. Variation within this range is largely dictated by the shape and size of the binding interface, with more convoluted and larger interfaces corresponding to lower basal rates whereas flatter and smaller interfaces corresponding to higher basal rates.

**Table 1:** Experimental and calculated rate constants for the association of seven actin-binding proteins with G-actin.

		Experiment			Calculation		
ABP	PDB	*k*_a_ (M^−1^ s^−1^)	*I* b (mM)	Ref.	*k*_a_ (M^−1^ s^−1^)	*k*_a0_ (M^−1^ s^−1^)	Δ*G*_el_* (kcal/mol)
Profilin	2BTF	1.4×10^7^	110	[5]	1.04×10^7^	3.60×10^5^	−1.99
Twinfilin ADF-H 2	3DAW	2.36×10^7^	110	[7]	1.22×10^7^	7.90×10^5^	−1.62
Gelsolin domain 1	1EQY	3.0×10^5^	16	[3]	2.00×10^5^	1.14×10^6^	1.03
WASP WCA	2A3Z	4.3×10^7^	65	[6]	1.65×10^7^	2.53×10^6^	−1.11
Ciboulot domain 1	1SQK	1.2×10^6^	10	[8]	0.87×10^6^	3.03×10^5^	−0.62
Tβ4	1SQK/1T44^a^	1.7×10^6^	5	[9]	4.04×10^6^	7.52×10^5^	−0.99
DBP	1KXP	2.2×10^4^	12	[4]	1.59×10^4^	7.42×10^4^	0.91

a The complex of Tβ4 with G-actin was modeled by merging PDB entries 1SQK and 1T44 (see Methods for details).

b Ionic strength.

The electrostatic contribution is determined by the degree of charge complementarity across the binding interface [2], [26]–[28]. G-actin is long known to have a largely anionic surface. Five of the seven ABPs studied here have G-actin binding sites largely decorated by cationic residues; the exceptions are gelsolin domain 1 and DBP (Supplementary Figure S1). The ABPs' different degrees of charge complementarity with G-actin result in the variation of the transient-complex electrostatic interaction energy (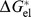) from 1.0 kcal/mol for gelsolin domain 1 to −2.0 kcal/mol for profilin (Table 1). The variation in 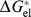 significantly widens the variation in *k*_a_, and can largely explain the three order of magnitude range of the observed *k*_a_ values.

Below we present the association mechanisms and rate constants of the seven ABPs in details.

### Profilin

The transient-complex based approach for computing the association rate constant consists of three components [25]: generation of the transient complex-ensemble; determination of the basal rate constant *k*_a0_ by Brownian dynamics simulations without any biasing force [29]; and calculation of the electrostatic interaction energy 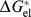 in the transient complex by solving the full Poisson-Boltzmann equation. Our algorithm for locating the transient complex is based on the following observation. The native-complex energy well is characterized by a large number of contacts (*N*_c_) between interaction loci across the interface but very restricted relative rotation between the proteins. Once outside the native-complex energy well the two proteins lose most of the specific short-range interactions while gaining nearly complete rotational freedom. The transient complex is then located at the midpoint of the sharp transition between these two regimes, where the value of *N*_c_ is denoted as 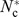 [10].

The profilin:G-actin native complex has a large, relatively flat interface, involving nearly the full base of G-actin (Figures 2B and S1A) [17]; 22 interaction loci on either side of the interface form 58 contacts. The transient complex is defined with 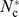 = 16 (Figure S2A). The calculated basal rate constant is 3.6×10^5^ M^−1^ s^−1^. The values of *N*_c_ in the native complex and in the transient complex are listed in Table 2 for easy comparison among the seven ABPs.

**Table 2:** Geometric and electrostatic properties of seven actin-binding proteins with G-actin.

	*N*_c_		Ion pairs^a^	
ABP	Native complex	Transient Complex	Attractive	Repulsive
Profilin	58	16	K69-D288; R74-Cterm; R88-E167; K90-D286, D288; K125-E361, E364; E82-K113, R372	K125-K373; D86-Cterm; E129-E364
Twinfilin ADF-H 2	44	13	Nterm-Cterm; R269-E334; K276, K294-E167; E296-R147; D298-R147, K328; E311-K291	E311-D292
Gelsolin domain 1	36	9		R96-R147
WASP residues 431–446	26	7	R431, R439-E167; K446-D25	R431-R116
Ciboulot residues 10–32	29	11	K19-E167; K31-D24	
Tβ4 residues 0–19	34	13	K3-E167; K14-E334; K18-D24; K19-D25	
DBP	72	17	K207-E167; R218-D288 E138-R147; E143-K328 E297, D298-K113	R203-R147

a Defined as any pair of charged residues that have a Coulomb interaction energy with a magnitude >13.8 kcal/mol (at dielectric constant = 4) in the native complex. This magnitude corresponds to a pair of unit charges at a distance of 6 Å. Residues before and after the dash are from the ABPs and G-actin, respectively.

Profilin and G-actin show a high degree of charge complementarity across the interface in the native complex (Figure S1A). Five cationic residues of profilin form ion pairs with seven anionic residues of G-actin (Table 2). Toward the back, the signs of the charges are reversed on both sides of the interface, with E82 of profilin paired with K113 and R372 of G-actin. Corresponding to the high degree of charge complementarity, the electrostatic interaction energy in the transient complex is −2.0 kcal/mol at ionic strength = 110 mM. Combining the contributions of the basal rate constant and the electrostatic interactions, the overall association rate constant is calculated to be 1.0×10^7^ M^−1^ s^−1^, which agrees well with the measured value of 1.4×10^7^ M^−1^ s^−1^ at the same ionic strength [5]. At a low ionic strength (5 mM), the measured rate constant is higher, at 4.5×10^7^ M^−1^ s^−1^ [13], [30]. We can explain the increase in *k*_a_ by a decrease in salt screening of the inter-protein electrostatic attraction. At the lower ionic strength, our calculations give 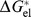 = −3.0 kcal/mol and *k*_a_ = 6.0×10^7^ M^−1^ s^−1^.

### Twinfilin C-terminal ADF homology domain

Twinfilin is comprised of two ADF homology domains. Both isolated domains bind to G-actin [7], but only the structure for the complex formed between ADF-H 2 and G-actin has been determined [21]. Relative to profilin, the binding site for twinfilin ADF-H 2 on G-actin is shifted from the base toward the front (Figure 2C). The interface is shaped like a slightly folded rectangle (to ∼130°), with the fold line, corresponding to the cleft-lying helix of twinfilin ADF-H 2, just off the diagonal of the rectangle (cf. Figure S1D–F). Interaction loci form 44 contacts across the interface. The transient complex is defined with 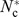 = 13, and the calculated basal rate constant is 7.9×10^5^ M^−1^ s^−1^.

Twinfilin ADF-H 2 also shows a high degree of charge complementarity with G-actin across the interface (Figure S1B). The binding site for twinfilin ADF-H 2 on G-actin is covered mostly by a negative electrostatic surface, delimited by E167 from the base side and E334 on the front side. A corner of this binding site, over subdomain 3, has a positive electrostatic surface, due to a cluster of cationic residues including R147, K291, and K328. Across the interface, twinfilin ADF-H 2 has a mostly positive electrostatic surface, delimited by K276 and K294 on the base side and R269 on the front side. The cationic corner of G-actin is paired with an anionic corner of twinfilin ADF-H 2, including E296, D298, and E311. The opposite charges form multiple ion pairs across the interface (Table 2). Correspondingly, the transient complex has a significant favorable electrostatic interaction energy: 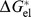 = −1.6 kcal/mol at an ionic strength of 110 mM. The resulting overall association rate constant is 1.2×10^7^ M^−1^ s^−1^, which agrees well with the measured value of 2.4×10^7^ M^−1^ s^−1^ at the same ionic strength [7].

### Gelsolin domain 1

Gelsolin is comprised of six homologous domains; domain 1 and domain 4 bind G-actin, whereas domain 2 binds to the side of F-actin [31]. Gelsolin domain 1 is structurally similar to twinfilin ADF-H 2, and their interfaces with G-actin are also very similar, except for a ∼20% reduction in interface area for the former ABP (Figures 2C, D and S1B, C). The number of inter-protein contacts is reduced commensurately from 44 for twinfilin ADF-H 2 to 36 for gelsolin domain 1. A similar reduction in 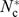, from 13 to 9, is obtained for the transient complex. Correspondingly there is a slightly increase in the basal rate constant, from 7.9×10^5^ M^−1^ s^−1^ for twinfilin ADF-H 2 to 1.1×10^6^ M^−1^ s^−1^ for gelsolin domain 1.

While the interfaces of twinfilin ADF-H 2 and gelsolin domain 1 with G-actin are structurally similar, the charge distributions of the two ABPs in their G-actin binding sites are almost the opposite of each other (Figure S1B, C). D85 and D86 of gelsolin domain 1 takes up the locations occupied by K276 and K294 of twinfilin ADF-H 2; the C-terminal carboxylate of the former swaps for R267 of the latter; and R96 of one exchanges for E296, D298 and E311 of the other. Consequently like charges are matched across the gelsolin domain 1:G-actin interface (Table 2), and the transient complex has a significant unfavorable electrostatic interaction energy. At an ionic strength of 16 mM, 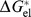 = 1.0 kcal/mol, resulting in overall association rate constant of 2.0×10^5^ M^−1^ s^−1^. This result matches well with the measured value of 3.0×10^5^ M^−1^ s^−1^ at the same ionic strength [3].

### WASP actin-regulatory region

WASP WCA (residues 431–502) can be further divided into the WASP homology 2 (WH2, also known as verprolin homology; residues 431–447) segment, linker (residues 448–461), central segment (residues 462–483), and acidic segment (residues 484–502) [23]. The WH2 and central segments bind G-actin [6], [23]; the central segment together with the acidic segment also binds Arp2/3 [6], [22], [23]. In the free state, WASP WCA is disordered [6], [22], [23]. Chereau et al. [20] determined the structure of a WASP peptide (residues 430–458) encompassing the WH2 segment and most of the linker. The WH2 segment consists of the cleft-lying helix and an extended C-terminal tail, whereas the linker portion is still disordered (Figure 2E). In the context of the full-length WASP in the free state, the central segment forms an amphipathic helix that has its nonpolar face docked to the GTPase binding domain (GBD), resulting in auto-inhibition of WASP [32]. Binding of a Rho-family GTPase, Cdc42, to the GBD releases the central segment, leading to the activation of WASP (Figure 1). In the complex with G-actin, the central segment is also likely to form an amphipathic helix that has its nonpolar face docked to G-actin [6], [23].

The likely binding site for the central-segment amphipathic helix is at the top of G-actin, in the cleft between subdomains 2 and 4 (Figure 2E). This is the site where a C-terminal helix of Tβ4 binds, as found in the structure of a gelsolin domain 1-Tβ4 chimera bound to G-actin [19] (see below). The distance between the C-terminus of the WH2 segment and the N-terminus of the central segment is then 30–35 Å, which is spanned by the 14-residue linker running along the nucleotide-binding cleft separating the subdomains 1 and 2 from subdomains 3 and 4 of G-actin.

We can model WASP WCA as a bivalent ligand, with the WH2 and central segments binding to separate sites on G-actin and connected by a linker. The equilibrium constant for simultaneous binding of the two segments can be written as [33]

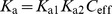
where *K*_a1_ and *K*_a2_ are the association constants for the two isolated segments, and *C*_eff_ is the effective concentration. If the linker is modeled as a worm-like chain that does not adversely affect the interactions of the two segments with their respective binding sites, then
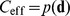
which is the probability density of the linker end-to-end vector when the latter is the displacement vector **d** from the C-terminus of the WH2 segment to the N-terminus of the central segment. The measured association constants are 3.2×10^5^ and 8.2×10^4^ M^−1^, respectively, for the isolated WH2 and central segments, and 3.1×10^6^ M^−1^ for WASP WCA [23]. The effective concentration calculated from the experimental association constants is 0.1 mM. In comparison, the value of *p*(**d**) calculated with the 14-residue linker modeled as a worm-like chain with *d* = 30–35 Å is 0.08–1 mM. So the linker model appears quantitatively reasonable.

**Figure 4:**
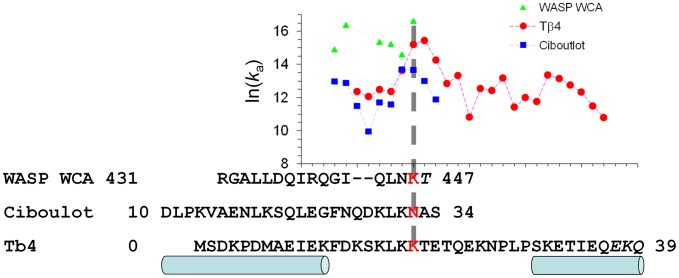
Identifying the docking segments for WASP WCA, ciboulot domain 1, and Tβ4 in their binding to G-actin, by finding the fragments with the highest rate constants for the docking step. The sequences of the three proteins are aligned. Regions forming helices are indicated by cylinders. In all three cases, the proposed docking segments are the fragments ending at the third position of the conserved “LKK” motif (ending residues shown in red). The corresponding rate constants for the docking step are indicated by a vertical dashed line. Inclusion of the C-terminal residues in italic in the proposed docking segments produced large gaps in *N*_c_, indicating that these residues must belong to the coalescing segments instead.

Given that WASP WCA is intrinsically disordered and forms an extended conformation on the surface of G-actin, it is unlikely that WASP WCA forms its contacts with G-actin all at once. It is more likely that the binding starts with the initial docking of one segment and continues with subsequent coalescing of another segment. This dock-and-coalesce mechanism formed the basis of calculating the association rate constants of intrinsically disordered proteins [10], [34]. The docking segment was identified with the one yielding the highest association rate constant, based on the following reasoning. First, multiple pathways could contribute to the binding, but the one yielding a much higher overall rate constant for forming the final complex than all alternative pathways would dominate. So we can just focus on the dominant pathway. Second, the coalescing step is likely to be fast so that the docking step becomes rate-limiting. So we can further narrow our consideration down to just the docking step, which allows for the treatment of our transient-complex based approach. In Figure 4 we display the rate constants calculated with six fragments of the WH2 segment proposed as the docking segment. The R431-K446 fragment gives the highest rate constant, 1.7×10^7^ M^−1^ s^−1^ (at ionic strength = 65 mM). This calculated result compares well with the measured rate constant, 4.3×10^7^ M^−1^ s^−1^ [6]. While the reasoning behind our approach seems well justified and the predicted *k*_a_ is validated by the experimental data, coarse-grained simulations of WASP WCA:G-actin association could yield direct evidence for the dock-and-coalesce mechanism.

We also modeled the structure of the WASP central segment bound to G-actin (see Methods for details). Based on this structure, our transient-complex based approach predicts a rate constant ∼10^4^ M^−1^ s^−1^. This is three orders of magnitude lower than the rate constant calculated with the WH2 fragment as the docking segment, thus justifying our contention that the dominant binding pathway of WASP WCA consists of the docking of the WH2 segment and the subsequent coalescence of the central segment (Figure 3B).

We now examine the physical determinants of the docking rate constant to provide a rationalization for its relatively high value, 1.7×10^7^ M^−1^ s^−1^. This value comes from a combination of a basal rate constant of 2.5×10^6^ M^−1^ s^−1^, the highest among all seven ABPs, and an electrostatic interaction energy 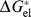 = −1.1 kcal/mol, the third most favorable. The high basal rate constant can be attributed to a relatively small interface (*N*_c_ = 26 in the native complex), formed by the docking of a 10-residue helix plus a six-residue extension to an open cleft (Figure S1D). With 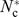 = 7, the transient complex is reached with relatively mild orientational restraints between the WASP WH2 segment and G-actin. The negative electrostatic surface over the cleft of G-actin has been noted above (also see Figure S1D). The WH2 segment complements this with a positive electrostatic surface facing the cleft. In particular, R431 at the start of the cleft-lying helix and K446 at the end of the C-terminal extension form ion pairs with E167 and D25 of G-actin, respectively (Table 2; Figure S1D). These favorable interactions explain the significant negative value of 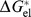.

### Ciboulot domain 1

Ciboulot domain 1 and Tβ4 are homologous, with sequence identities of 25% and 58%, respectively, for the N-terminal half (ciboulot domain 1 residues 14–33 and Tβ4 residues 1–20) and C-terminal half (ciboulot domain 1 residues 34–52 and Tβ4 residues 21–39). Several lines of evidence suggest that the binding of ciboulot domains 1 (and Tβ4) to G-actin also follows the dock-and-coalesce mechanism, with the N-terminal half as the docking segment and the C-terminal half as the coalescing segment. First, like WASP WCA, these two proteins are intrinsically disordered and adopt extended conformations upon binding G-actin [8], [24]. Second, in the crystal structure of the complex with G-actin, the N-terminal half of the ciboulot domain 1 is resolved whereas the C-terminal half is still disordered (Figure 2F) [8]. Third, X-ray scattering data of the ciboulot domain 1:G-actin complex at low ionic strength could be fitted with the N-terminal and C-terminal halves bound to the barbed end and pointed end of G-actin, respectively, but not the data at physiological ionic strength [35]. The latter data was consistent with a model in which the N-terminal half is bound but the C-terminal half is dissociated. Fourth, for G-actin-bound ciboulot domain 1, ^1^H-^15^N NMR cross peaks of the C-terminal half disappeared or attenuated upon a temperature increase from 25°C to 35°C, indicating either dissociation from or weakened interactions with G-actin [8]. Finally, ciboulot-bound G-actin must have its pointed end free for it to be competent for filament growth at the barbed end.

Using the transient-complex based approach, we calculated the rate constants of the docking step with 10 fragments of the N-terminal half as the possible docking segment (Figure 4). The D10-N32 fragment gives the highest rate constant. The value at low ionic strength (10 mM), 0.9×10^6^ M^−1^ s^−1^, is close to the measured rate constant, 1.2×10^6^ M^−1^ s^−1^ [8]. We note that ciboulot N32 aligns to WASP K446, which is the last residue of the putative docking segment of WASP WCA.

For the docking of the D10-N32 fragment, the basal rate constant is 0.3×10^6^ M^−1^ s^−1^ and the electrostatic interaction energy 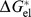 is −0.6 kcal/mol. This basal rate constant is 10-fold lower than that for docking the corresponding WASP fragment, due to additional contacts. In ciboulot, the cleft-lying helix is longer by 1 turn of helix at the N-terminus, and the sequence linking this helix and the conserved “LKK” motif (_30_LKN_32_ in ciboulot and _444_LNK_446_ in WASP) is longer by two residues (Figure 4). The transient complex for the ciboulot fragment is defined with 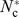 = 11 (Figure S2B), an increase of four contacts from the WASP counterpart. Ion pairs with G-actin are maintained at the start and end of the ciboulot fragment (Table 2), but the electrostatic surface at the end of the fragment is not as strongly positive as that of the WASP fragment (Figure S1E). This accounts for the moderation in 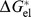 relative to WASP.

### Thymosin β4

Unlike ciboulot domain 1, Tβ4 sequesters G-actin and prevents its addition to the barbed end of actin filaments. This difference can largely be explained by a higher affinity of the Tβ4 C-terminal half, relative to the ciboulot domain 1 counterpart, for the pointed end of G-actin [8], [35]. Nevertheless the complex formation with G-actin by Tβ4, with both the N-terminal and C-terminal halves bound, is expected to follow the same dock-and-coalesce mechanism as ciboulot domain 1. Didry et al. [35] designed a chimera by combining the N-terminal half of ciboulot domain 1 and the C-terminal half of Tβ4. The change in intensities of ^1^H-^15^N NMR cross peaks suggested that the C-terminal half of the chimera became dissociated upon raising the ionic strength from low to physiological range. Even at low ionic strength, exchange between unbound and bound states on the 10-ms timescale was observed in NMR experiments for residues in the C-terminal half of the chimera.

Following Irobi et al. [19], we built the structure of Tβ4 bound to G-actin by combining models for the first 16 residues and for residues 17–39 (Figure 2G). The N-terminal portion was a homology model based on the G-actin-bound ciboulot domain 1 [8], and the C-terminal portion was taken from the structure of the gelsolin domain 1-Tβ4 chimera bound to G-actin [19]. This structural model for the full-length Tβ4 allowed us to exhaustively search for the docking segment in implementing the dock-and-coalesce mechanism. As explained above, the docking segment is selected to yield the highest rate constant for the docking step.

Figure 4 displays the rate constants for the docking step calculated with fragments ending at residues 14 to 36 (at ionic strength = 5 mM). The highest rate constant, 5.2×10^6^ M^−1^ s^−1^, is for the fragment ending at residue T20, and a very close second, at 4.0×10^6^ M^−1^ s^−1^, is obtained for the fragment with one less residue, ending at K19. That residue aligns with the last residues of the docking segments obtained above for the G-actin binding of WASP WCA and ciboulot domain 1. To maintain consistency among the three proteins, we propose the fragment ending at K19 as the docking segment for Tβ4. The calculated rate constant, 4.0×10^6^ M^−1^ s^−1^, for the docking step only slightly overestimates the overall association rate constant measured at the same low ionic strength [9].

The higher rate constants for the fragments ending at K19 and T20 relatively to those for shorter and longer fragments can largely be attributed to differences in 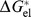. In particular, K18 and K19 make very strong favorable electrostatic interactions with G-actin D24 and D25, respectively (Table 2; Figure S1F). The transient complex of our proposed docking segment (residues M0 to K19) has 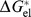 = −1.0 kcal/mol (at 5 mM ionic strength). For shorter fragments, 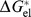 is positive. For increasingly longer fragments, 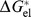 first has a diminished magnitude and then switches the sign to positive.

To further highlight the electrostatic contribution of K18 and K19, we neutralized them by mutation to alanine. The mutant docking segment has 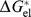 = 0.7 kcal/mol. Correspondingly the calculated rate constant reduces by 19-fold to 2.1×10^5^ M^−1^ s^−1^. This compares favorably with the measured 10-fold reduction in the G-actin binding rate constant of the Tβ4 K18A/K19A mutant [9].

### Vitamin-D binding protein

DBP sequesters G-actin in the plasma. Its three domains tightly clamp around the barbed end of G-actin (Figure 2H). The structure of DBP in the free state, while slightly more open [18], is still too narrow for G-actin to enter. We used normal mode analysis based on an elastic network model [36] to mimic the breathing motion that DBP likely undergoes during the association process (Figure 3C). The lowest-frequency mode of DBP would widen the opening between domains 1 and 3 (along with a shear motion between the two domains; Figure S3), yielding a transient “open” conformation that is ready for binding G-actin.

We treated the structure in which G-actin is bound to the transient open conformation of DBP as the native complex and applied the transient-complex based approach to calculate the association rate constant. The result, 1.6×10^4^ M^−1^ s^−1^, agrees well with the measured value of 2.2×10^4^ M^−1^ s^−1^ (at ionic strength = 12 mM) [4]. The calculated rate constant came from a low basal rate constant of 7.4×10^4^ M^−1^ s^−1^ and an unfavorable electrostatic interaction 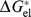 = 0.9 kcal/mol. The G-actin binding site on DBP is shaped like a scoop, with a deep basin for the base of G-actin. The large interface area (*N*_c_ = 72 in the native complex) along with the highly curved shape results in a very restricted transient complex (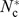 = 17; Figure S2C), accounting for the low basal rate constant.

The unfavorable 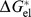 is somewhat unusual, since the DBP:G-actin interface features multiple attractive ion pairs (Table 2). The overall positive 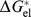 can be explained by the large net negative charges carried by both proteins (net charges of −13 and −12, respectively, for DBP and G-actin; see Figure S1G). Engulfing of G-actin by the three domains of DBP ensures that the numerous anionic residues on the two proteins are not very distant. Their repulsion trumps the attraction of the ion pairs in the interface.

The transient open conformation of DBP used in the *k*_a_ calculation was from a 200-ps molecular dynamics refinement of a structure along a normal mode (see Methods for details). To get some sense on how conformational dynamics may affect the calculation results, we also carried out *k*_a_ calculations on the snapshots at 100 and 150 ps of the refinement. Compared to a value of 17 for the 200-ps snapshot, 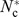 decreases to 15 and 10 for 100- and 150-ps snapshots, respectively. Correspondingly, *k*_a0_ increases from 7.4×10^4^ M^−1^ s^−1^ to 1.5×10^5^ and 2.6×10^5^ M^−1^ s^−1^, and 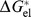 increases from 0.9 kcal/mol to 1.3 and 1.4 kcal/mol, respectively. As a result of the compensatory changes in *k*_a0_ and 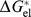, the overall rate constant is unchanged for the 100-ps snapshot (*k*_a_ still at 1.6×10^4^ M^−1^ s^−1^) and minimally changed for the 150-ps snapshot (*k*_a_ = 2.4×10^4^ M^−1^ s^−1^). So all the snapshots from the molecular dynamics refinement give essentially the same *k*_a_.

## Discussion

We have carried out rate calculations for the association of seven ABPs with G-actin, and provided quantitative rationalization for the 1000-fold rate variations among the seven ABPs. The results demonstrate that ABPs can use their physical properties, in particular molecular flexibility and surface charges, in a variety of ways to modulate both the mechanisms of association and the magnitudes of association rate constants.

The widely-varying rate constants of the ABPs appear to be tuned for their distinct regulatory functions. WASP WCA has the highest association rate constant, and this is fitting because WASP's recruitment of G-actin to the Arp2/3 complex is critical for the nucleation of new filaments. Deleting the WH2 segment, which according to our calculation is responsible for the high association rate constant with G-actin, leads to a significant slowing down of actin polymerization [23]. Profin and twinfilin, both having rate constants exceeding 10^7^ M^−1^ s^−1^, are responsible, respectively, for sequestering G-actin newly dissociated from the pointed end of filaments and for bringing G-actin to the barbed end for filament growth. Rapid G-actin association by these two ABPs would allow for rapid remodeling of actin cytoskeleton in response to external stimuli. The rate constant of DBP is the lowest. Presumably, tight, not rapid, binding of G-actin is key to ensure the disassembly of potentially lethal actin filaments in the bloodstream. Spontaneous nucleation of ADP-G-actin is extremely slow [37], so DBP:G-actin association faces only poor potential kinetic competition.

The seven ABPs exhibit three classes of association mechanisms (Figure 3), dictated by molecular shapes and flexibility. Relatively rigid globular proteins like profilin can reach the transient complex with G-actin by diffusion and then rapidly form their specific interactions nearly all at once. In contrast, an intrinsically disordered protein that adopts an extended conformation in the native complex with its target is unlikely to form their specific interactions all at once. It is more likely different segments of the protein contact the target at different times. In principle multiple pathways, each with the different segments contacting the target in a specific sequence, can lead to the native complex. Often one pathway dominates, leading to the dock-and-coalesce mechanism. This apparently is the case for the binding of WASP WCA, ciboulot domain 1, and Tβ4 to G-actin. Finally, the fork-shaped DBP has an opening that is too narrow, both before and after G-actin binding, for G-actin to enter. Therefore DBP must make excursions to conformations with a wider opening before G-actin can enter. In the usual sense, the last two mechanisms would be referred to as induced folding and conformational selection, respectively. The diverse shapes/flexibility and association mechanisms of the ABPs provide a nice illustration of “form dictates function.”

Regardless of the association mechanism, the association rate constants can span a wide range. Although the ABP (i.e., DBP) that follows the transient-opening mechanism in the present study has the lowest association rate constant, our previous study [10] identified the association of ribonuclease A with ribonuclease inhibitor as following the same mechanism, and yet the association rate constant in this case is as high as 3.4×10^8^ M^−1^ s^−1^ [38]. So the association mechanism does not dictate the association rate constant. Rather, according to our transient-complex theory [10], [25], the association rate constant is determined by the basal rate constant, modeling the approach to the transient complex by free diffusion, and the electrostatic interaction energy in the transient complex. The basal rate constant usually falls in the range of 10^4^ to 10^6^ M^−1^ s^−1^ [10] and the variation is determined by the extent of orientational restraints between the proteins in the transient complex [39]–[41]. The extent of orientational restraints can be traced to the shape and size of the binding interface, and seems to be captured well by 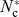, the number of contacts in the transient complex. There is good anti-correlation between ln(*k*_a0_) and 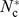 (Figure S4). In particular, the binding interface of DBP with G-actin has a large area and a highly curved shape. Correspondingly, DBP has the highest 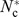 and lowest *k*_a0_. That low *k*_a0_ contributes to the low overall association rate constant of DBP. The electrostatic interaction energy in the transient complex can modulate the association rate constant by over four orders of magnitude [42] and largely explains the wide variation in rate constant among the seven ABPs studied. Its sign and magnitude are determined by the amount of charges carried by the proteins and degree of their complementarity across the interface [2], [26]–[28].

The seven proteins studied here all bind to the same site on the same protein, yet they exhibit such diversity in association mechanisms and wide variation in rate constants. Dissecting the physical determinants of this diversity in association kinetics has now provided better insight into how the different structures of the ABPs allow them to achieve their distinct regulatory functions. The structures for two other proteins bound to G-actin are found in the Protein Data Bank (PDB). A RPEL motif from the serum response factor coactivator MAL forms two helices on the G-actin surface, in a location similar to those occupied by the seven ABPs (PDB entry 2V52) [43]. The N-terminal helix lies in the cleft between subdomains 1 and 3, and the C-terminal helix interacts with subdomain 3 at the base. On the other hand, DNase I binds to G-actin at its top, interacting predominantly with subdomain 2 but also with subdomain 4 (PDB entry 2A42) [20]. We predict association rate constants of 5.8×10^5^ and 6.6×10^5^ M^−1^ s^−1^, respectively, for these two complexes. These results await experimental verification.

Many other ABPs have yet to have structures determined for their complexes with actin. Some of these structures, including those for G-actin-bound complexes of ADF and of twinfilin ADF-homology domain 1, can be modeled. Application of our transient-complex based approach for characterizing association kinetics to these new targets will further advance our understanding of how ABPs regulate actin dynamics.

## Methods

### Structure preparation for native complexes

The input to our transient-complex based approach for calculation protein association rate constants is the structures of native complexes. For the G-actin-bound complexes of profilin, twinfilin ADF-homology domain 2, and gelsolin domain 1, we directly used the structures of PDB entries 2BTF [17], 3DAW [21], and 1EQY [16], respectively. A calcium ion coordinated by D85 carboxyl oxygens and G90 and A92 carbonyl oxygens of gelsolin domain 1 as well as an E167 carboxyl oxygen of G-actin was included as part of the gelsolin molecule. All hydrogen atoms were added and energy minimized by the AMBER program.

The structures of the G-actin-bound complexes of the WASP WH2 segment and ciboulot domain 1 were from PDB entries 2A3Z [20] and 1SQK [8], respectively. The C-terminal portions of the two ABPs were trimmed to various extents to produce putative docking segments for rate calculations. The structure of Tβ4 bound to G-actin was built according to Irobi et al. [19]. The N-terminal 16-residue portion was a homology model using PDB entry 1SQK (the G-actin-bound ciboulot domain 1) [8], with the sequences aligned according to Figure 4. The C-terminal 23-residue portion was taken from PDB entry 1T44, which is the structure of a gelsolin domain 1-Tβ4 chimera bound to G-actin [19]. The two portions were merged after superimposing the G-actin molecules in the two parent structures. The Tβ4 sidechains were then refined by energy minimization.

The structure of the WASP central segment (residues S462–S479) bound to G-actin was modeled as follows. The initial conformation of this segment, taken from the auto-inhibited structure (PDB entry 1EJ5 [32]). The amphipathic helix in this segment was aligned to the C-terminal helix of Tβ4 bound to G-actin [19] such that five WASP residues implicated as being buried in the interface with G-actin by NMR experiments [23] were positioned toward G-actin. The three N-terminal residues preceding the amphipathic helix was manually adjusted to roughly follow the corresponding residues in the Tβ4 structure. The G-actin-bound complex of the WASP central segment was then refined by energy minimization and molecular dynamics simulation for 100 ps in explicit solvent.

The initial structure for the G-actin-bound complex of vitamin-D binding protein was from PDB entry 1KXP [18]. The open DBP conformation was built on the lowest-frequency normal mode (obtained by running the ElNemo program [36]). The amplitude of the motion along this mode was set with DQ = 300. G-actin was then brought back and the complex was refined by energy minimized and molecular dynamics simulation for 200 ps in explicit solvent.

### Rate calculation by the transient-complex based approach

Our transient-complex based approach for calculating protein-protein association rate constants has been described previously [25] and has been implemented into a web server (http://pipe.sc.fsu.edu/transcomp/) [10]. All the *k*_a_ calculations reported here were done via the TransComp server, without human interrogation. TransComp consists of three steps. The first is the generation of the transient complex, the late intermediate located at the rim of the native-complex energy well, by generating configurations of two associating proteins around the native complex. In the native-complex energy well the two proteins have a large number of contacts, *N*_c_, between interaction loci across the interface but a small standard deviation, *σ_χ_*, in the values of the relative rotation angle *χ* in the sampled configurations (Figure S2). As the two proteins move outside the native-complex energy well they immediately gain nearly full freedom in relative rotation. Hence there is a sharp increase in *σ_χ_* as *N*_c_ is decreased. We fit the dependence of *σ_χ_* on *N*_c_ to a function used for modeling protein denaturation data as a two-state transition, and identify the midpoint of the transition, where *N*_c_ is denoted as 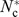, as the transient complex.

Once the transient complex is determined, the second step is to calculate the basal rate constant *k*_a0_ from Brownian dynamics simulations, using an algorithm developed previously [29]. In these simulations, there is no force or torque acting on the diffusing proteins, except when they encounter steric clash, which is treated as a reflecting boundary condition. The reaction surface (i.e., condition for forming the native complex) is identified as the transient-complex ensemble (i.e., *N*_c_ = 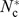). The third step is to calculate the electrostatic interaction energy 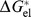 in the transient-complex ensemble, by solving the full, nonlinear Poisson-Boltzmann equation by the APBS program [44].

The transient-complex based approach treats the associating proteins as rigid. In some cases reaching the native complex by rigid-body docking always encounters steric clashes. Then there would be a large gap in the values of *N*_c_ calculated from the sampled configurations. The gap in *N*_c_ indicates that at least one of the proteins must be flexible or undergo conformational fluctuation during the association process. In particular, large gaps were found in docking the WASP R431-T447 fragment to G-actin and in the G-actin association of DBP using either the free or bound conformation. As we propose here, only a segment of WASP WCA first docks to G-actin; the C-terminal portion subsequently coalesces to its sub-site on G-actin. And DBP first undergoes breathing motion to produce an open conformation before G-actin enters.

## Supporting Information

Figure S1 Electrostatic surfaces of seven ABPs and their G-actin partners. (A) Profilin. (B) Twinfilin ADF-H 2. (C) Gelsolin domain 1. (D) WASP docking segment (residues 431–446). (E) Ciboulot docking segment (residues 10–32). (F) Tb4 docking segment (residues 0–19). (G) DBP. The left panels show G-actin in electrostatic surface and the ABPs in green ribbon, with the viewer looking into the ABP binding sites on G-actin from the side of the ABPs; the right panels have the representations and the viewing direction both reversed. G-actin always has its subdomain 3 at the top and subdomain 1 at the bottom. In (A) and (G), G-actin molecules are in the same orientation, with the G-actin base in front view; the orientation of G-actin molecules in (B)–(F) is rotated by 70°, placing the front of G-actin in the viewing direction.
(TIF)

Figure S2Locating the transient complex. (A) Profilin. (B) Ciboulot domain 1. (C) DBP. *χ* is the rotation angle between an ABP and G-actin in configurations sampled around the native complex; *σ_χ_* is the standard deviation in *χ* of configurations with a given contact number (*N*_c_). Left panels display *χ* vs. *N*_c_ scatter plots of sampled configurations; right panels display the dependence of *σ_χ_* on *N*_c_ and its fit to a function used for modeling protein denaturation data as two-state transition. The midpoint of the transition, where *N*_c_ is designated 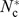, identifies the transient complex.(TIF)

Figure S3 Lowest-frequency normal mode of vitamin-D binding protein. Arrows indicate the relative amplitudes and directions for the motions of individual residues of DBP. The subdomains of G-actin are represented in the same coloring scheme as in Figure 2A; its orientation is the same as in Figure 3C. G-actin subdomains 3 and 4 are in the foreground (located at the bottom and top, respectively); domains 1 and 3 of DBP are on the right and left, respectively.
(TIF)

Figure S4Correlation between ln(*k*_a0_) and 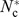. Data for the seven ABPs are shown as circles. Results of a linear regression analysis are shown.(TIF)
