# Supplementary figures and images for: Prediction and Dissection of Widely-Varying Association Rate Constants of Actin-Binding Proteins
Source: PLoS Comput Biol. 2012 Oct 4;8(10):e1002696. doi: 10.1371/journal.pcbi.1002696 (PMC3464195; doi:10.1371/journal.pcbi.1002696)

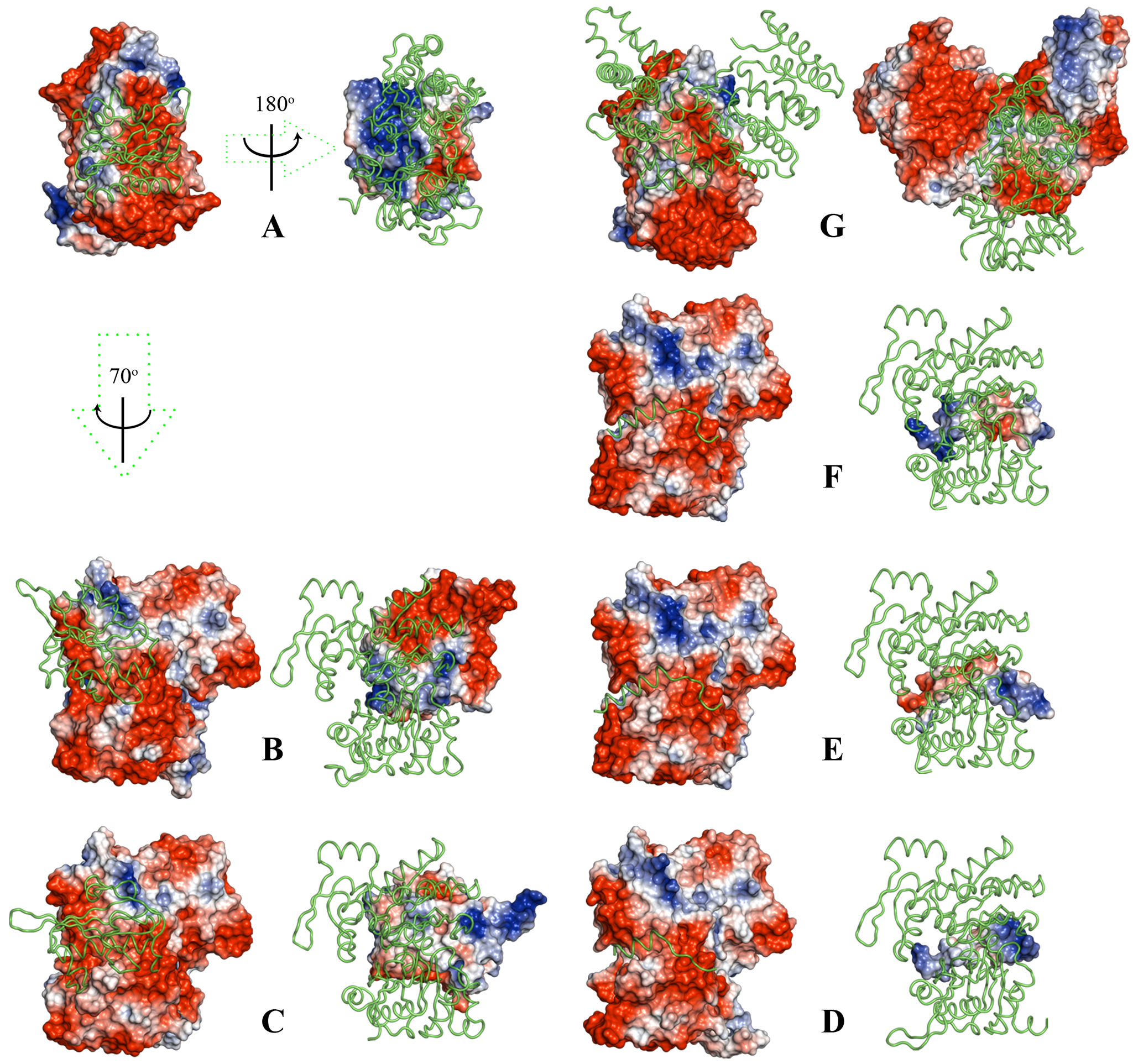

Supplement: Figure S1 — Electrostatic surfaces of seven ABPs and their G-actin partners. (A) Profilin. (B) Twinfilin ADF-H 2. (C) Gelsolin domain 1. (D) WASP docking segment (residues 431–446). (E) Ciboulot docking segment (residues 10–32). (F) Tb4 docking segment (residues 0–19). (G) DBP. The left panels show G-actin in electrostatic surface and the ABPs in green ribbon, with the viewer looking into the ABP binding sites on G-actin from the side of the ABPs; the right panels have the representations and the viewing direction both reversed. G-actin always has its subdomain 3 at the top and subdomain 1 at the bottom. In (A) and (G), G-actin molecules are in the same orientation, with the G-actin base in front view; the orientation of G-actin molecules in (B)–(F) is rotated by 70°, placing the front of G-actin in the viewing direction. (TIF) [file pcbi.1002696.s001.tif]

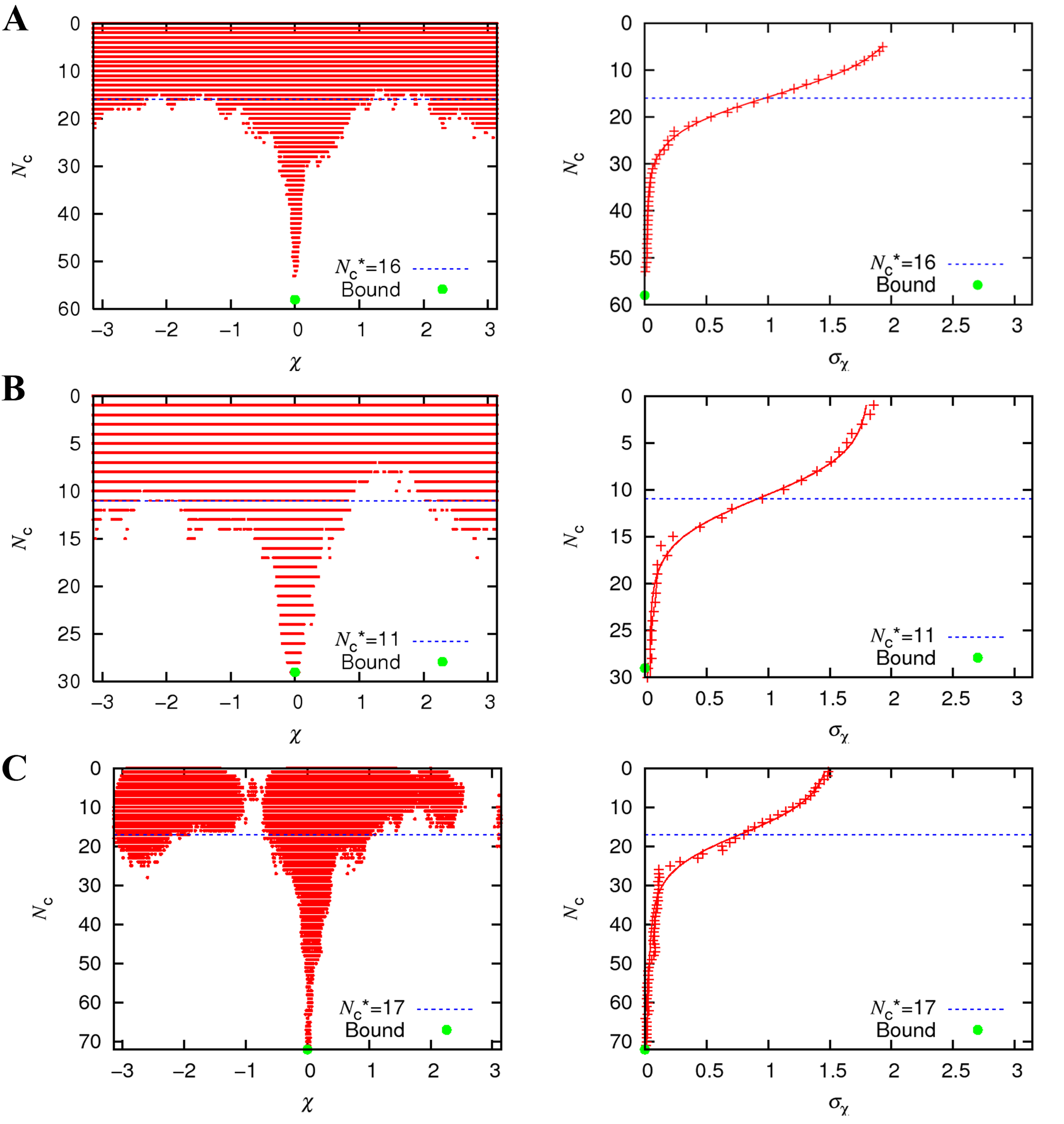

Supplement: Figure S2 — Locating the transient complex. (A) Profilin. (B) Ciboulot domain 1. (C) DBP. χ is the rotation angle between an ABP and G-actin in configurations sampled around the native complex; σχ is the standard deviation in χ of configurations with a given contact number (N c). Left panels display χ vs. N c scatter plots of sampled configurations; right panels display the dependence of σχ on N c and its fit to a function used for modeling protein denaturation data as two-state transition. The midpoint of the transition, where N c is designated , identifies the transient complex. (TIF) [file pcbi.1002696.s002.tif]

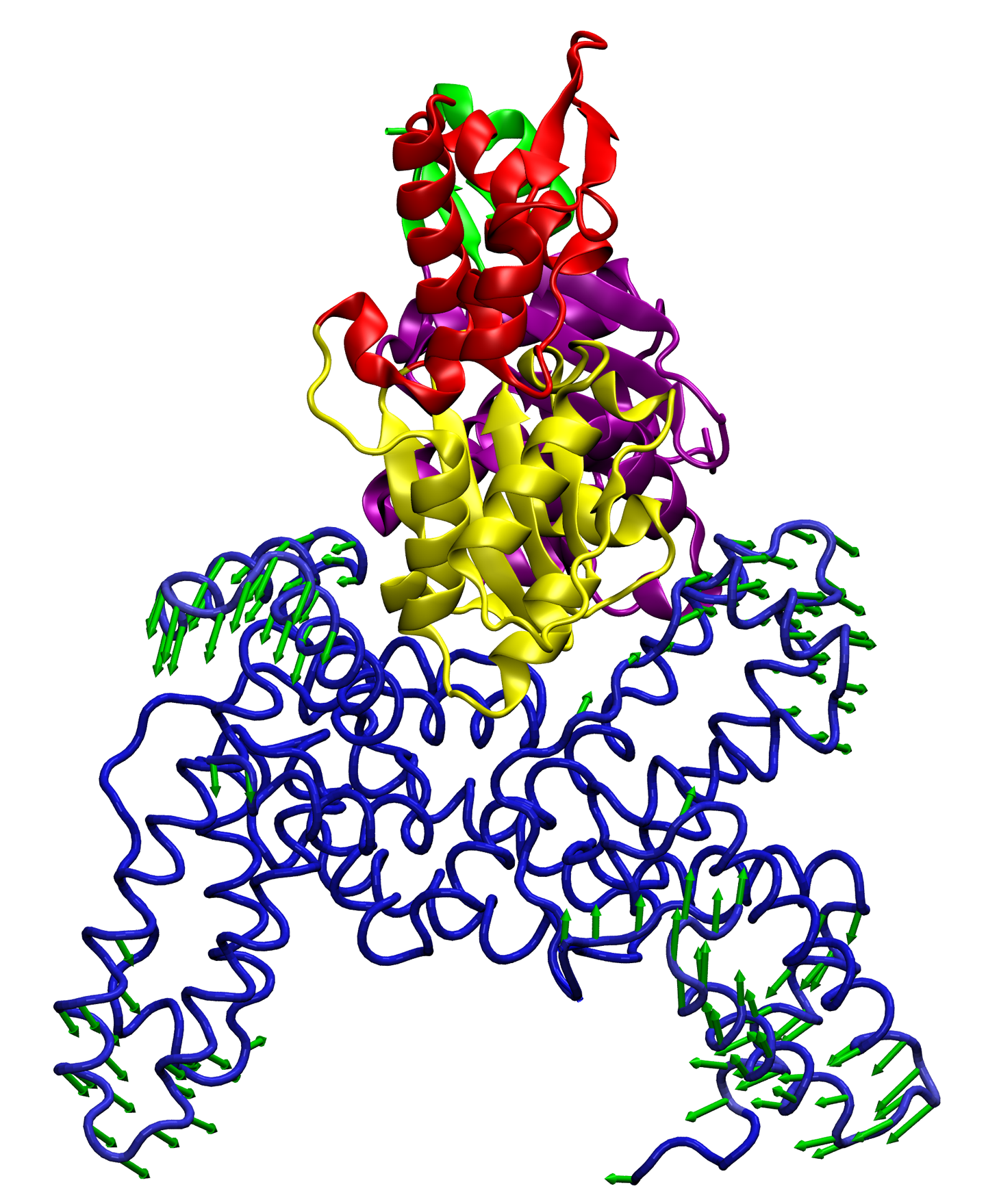

Supplement: Figure S3 — Lowest-frequency normal mode of vitamin-D binding protein. Arrows indicate the relative amplitudes and directions for the motions of individual residues of DBP. The subdomains of G-actin are represented in the same coloring scheme as in Figure 2A; its orientation is the same as in Figure 3C. G-actin subdomains 3 and 4 are in the foreground (located at the bottom and top, respectively); domains 1 and 3 of DBP are on the right and left, respectively. (TIF) [file pcbi.1002696.s003.tif]

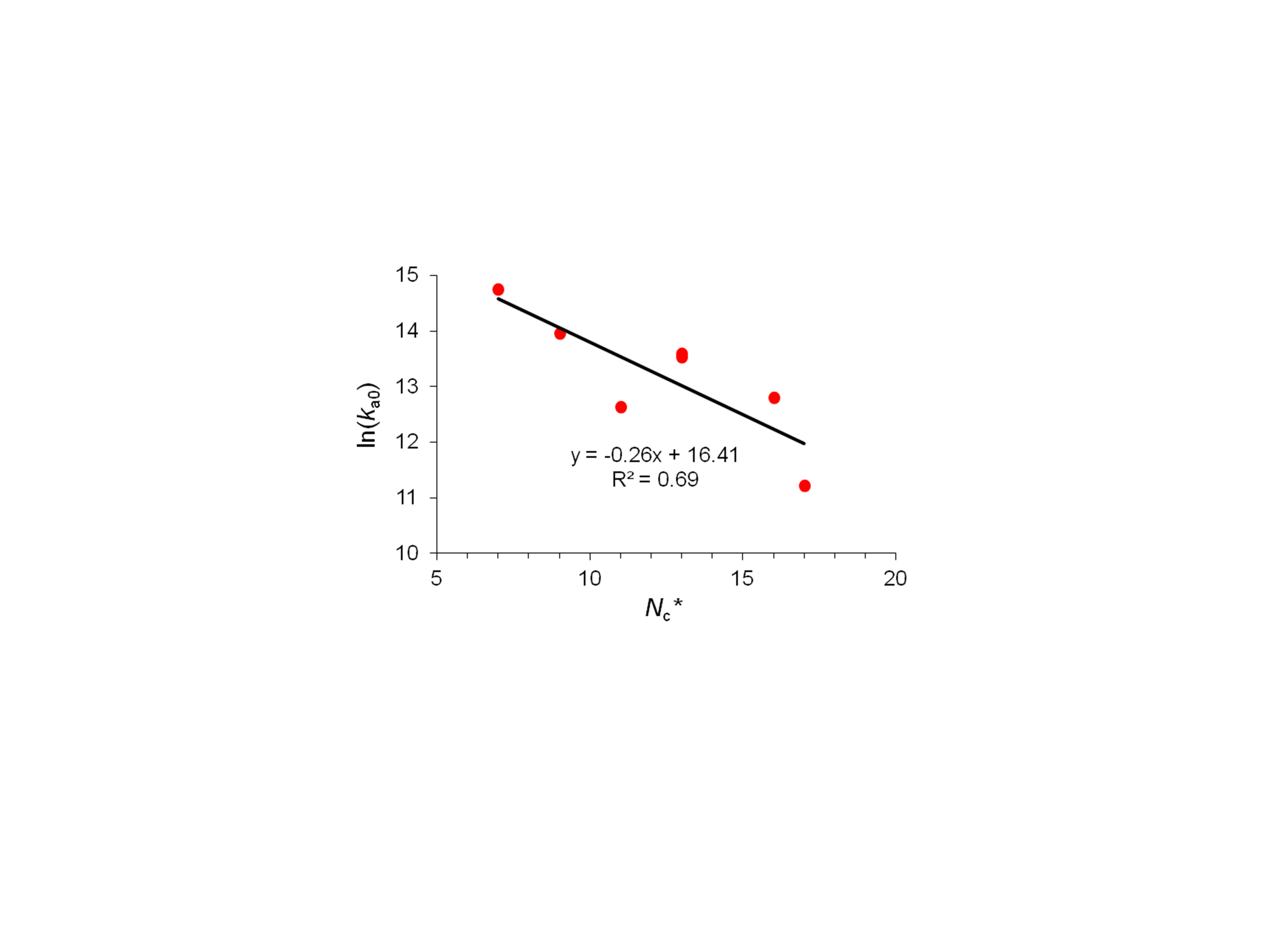

Supplement: Figure S4 — Correlation between ln(k a0) and . Data for the seven ABPs are shown as circles. Results of a linear regression analysis are shown. (TIF) [file pcbi.1002696.s004.tif]
